# The Chemistry of Dental Caries

**Published:** 1884-09

**Authors:** Wm. C. Wardlaw


ARTICLE III.

### THE CHEMISTRY OF CARIES.

BY W. C. WARDLAW, M. D., D. D. S.

[Read before the Georgia State Dental Society, May 24th, 1884.]

Caries is, perhaps, the most universal ill to which flesh is heir, and the importance of the study of its chemistry is conceded by the entire dental profession. Its ravages, and the advance of science alike, forbid content with the knowledge hitherto attained, but urge still further progress in search of curative treatment and preventitive measures. The fixed routine, tread-mill practice of our fathers, of stuffing teeth with gold, tin, &c., will not do; we must look for the initial cause of this trouble, which antagonizes us, and as we become better acquainted with its peculiar characteristics, we will be able the more intelligently to combat it.

Chemistry being, next to mathematics, the most exact of the sciences, it might seem at first blush an easy matter for the practical chemist to take tooth-substance and acids, and by a system of experimentation, classify the varieties of caries and determine the respective acid producing each. But the oral cavity contains such a conglomeration of matters, and presents such a concatenation of modifying circumstances, that the numerous chemical reactions are distinguishable only by the most scientific expert. Making no pretension to any such qualifications, I can in this paper only travel over the ground surveyed by others, and endeavor to clothe their deductions in such language as may by its novelty, commend them to the minds of some of you. In doing this, I am naturally inclined to follow in the track of that great pioneer in dental chemistry, Dr. George Watt, of Xenia, Ohio, although Miller andothers are not in perfect accord with him.

The various theories of caries have been so closely studied and so warmly discussed by their respective advocates, that one after another has been eliminated, until now there seems to be but two urging their rival claims before the profession, the acid and the germ (bug) theory. We freely admit the presence, and possibly active, influence of bacteria in the progress of caries; but that they are the prime and potent factor in the case, recent investigations seem to disprove. Miller, of Berlin, has most exhaustively and scientifically studied this question, and has come to this conclusion.

The visible effects of caries show it to be a process in which tooth-substance is disintegrated, broken down and washed away. This dissolution can be experimentally duplicated within and without the mouth, through the agency of acids. We therefore assume the truth of the acid theory, and proceed to investigate its "rationale."

The teeth are composed of organic and inorganic, or animal or mineral matters, held together by the powers of cohesion and chemical affinity, and acted upon, as all substances are, by mechanical and chemical forces. The constituents of enamel are inorganic mostly, phosphate of lime (bone phosphate) and carbonate of lime constituting about ninety-five per cent. Dentine, though having more animal matter, is composed of like elements in less amounts. The mineral acids readily unite with these earthly bases, forming salts of various degrees of solubility. If, therefore, we take such acids as sulphuric, hydrochloric and nitric, having this affinity for these bases, and place them in contact with enamel or dentine, chemical action at once supervenes, the strongest affinity asserting itself, and new compounds are evolved.

It is well here to bear in mind that chemical action is definite and specific. There is no uncertainty, like combinations always produce like results. One element combines with another element and produces a third substance; always, that, and never another. Hence, we should be able to prove our position, both by analysis and synthesis. We should, as the lawyers say in regard to circumstantial evidence, be able to establish our theory upon this supposition to the reasonable exclusion of all others.

The action of acids upon the teeth are modified by their concentration or dilution of the organization of the tooth, and by the resistance of vitality. Thus, if a dilute acid is placed in contact with dense, tooth-substance, it acts slowly upon it, making careful selection, however, of the particular element with which to combine; and "vice versa." A well-organized tooth, too, is of course more resistive than one with seams and cracks and poorly calcified materials. With these, and other modifying influences duly considered, a carefully conducted series of experiments would ascertain for us the specific effect following the contact of the various acids with the teeth. And such has been done for us.

Sulphuric acid, e. g., has such affinity for water, that it will decompose most organic substances (composed of oxygen, hydrogen and carbon), in order to gratify its desire, leaving dark traces of its foot-prints in the uncombined element, carbon. If, by means of a crack in the enamel, or a poorly calcified spot, or a rough point on a tooth, sulphuric acid, dilute or concentrated, gets a hold upon the tooth, the enamel is gradually disintegrated, the sulphuric acid uniting with the carbonate of lime, leaving the animal matter discolored. Dr. Watt maintains that this is the agent causing the dark appearance in that species of caries known as "black decay," which is generally superficial, slow in progress, and of a dark, often polished, appearance.

He says, that in this variety the organic matter is blackened, or carbonized, but there is only slight disintegration, of tooth-substance; just the effect we would expect to observe, where the sulphuric acid unites with the carbonate of lime, which is protected, as it were, by the more abundant and insoluble phosphate of lime circumjacent. There is no difficulty in accounting for the presence of this acid in the month, because the always-abundant supply of sulphurette hydrogen is readily oxydized, affording water and sulphuric acid.

To explain rather more fully: The constituents of the enamel, animal matter, carbonate of lime, and phosphate of lime, are commingled throughout. The phosphate of lime being in excess and not decomposed, the acid cannot penetrate very deeply into it, and it therefore protects, in a measure, the carbonate of lime from destruction, and serves to maintain the shape of the part.

The animal matter is not entirely decomposed either, but is carbonized; that is, its oxygen and hydrogen go to form water, which is greedily seized upon by the sulphuric acid, leaving the carbon in its natural blackness. Hence we see the disintegration is small, the decay is slow, and the color is such as to give the name of "black" decay. Sulphuric acid will give results similiar to these outside the mouth as well, and no other acid will, hence the reasonable conclusion that it is the destructive agent in "black decay."

Proceeding from the decidedly black decay, we find the color gradually becoming lighter, until we reach that generally denominated "brown decay."

Let us next examinine its physical characteristics, and look for the chemical agent, which has been at work to produce them.

Here the color, in the well-marked type, is brownish, the decay is more penetrating, the progress is more rapid, and the residum is of a semi solid, tough, leathery consistency.

Evidently the earthy constituents have been the first dissolved, and then washed away, leaving the organic, or animal matter partly dissolved and in process of fermentation and putrefaction. Hydrochloric acid has been getting in its work here, and something after this manner. The carbonate of lime and the hydrochloric acid are mutually decomposed, the results being water, carbonic acid and chloride of lime. This chloride of lime is a very soluble salt, and is readily washed away by the fluids of the mouth, whilst the carbonic acid escapes as gas. The phosphate of lime is also decomposed, forming a soluble compound, which also being removed, we have left the animal tissue almost untouched; hence the leathery layers of sensitive matter.

This action is well illustrated in the common experiment by the anatomist, where the lime-salts of a bone is dissolved away by hydrochloric acid, leaving the gelatinous portion untouched and still preserving the original form of bone. I have seen the humerus tied into a knot after having been so treated. Now, does the mouth furnish the conditions necessary for the formation of this powerful mineral acid? It certainly does. The great affinity of chlorine for oxygen results in the formation of hydrochloric acid, when ever they are brought within the spere of their affinities. The one article of chloride sodium (common salt) so freely used as a condiment, and which too is always found as a constituent of the saliva, abundantly furnishes the necessary chlorine to the expectant oxygen.

And next we have to face that other foe, which practical experience has taught us so much to dread—"white decay."

Nitric acid, we believe, may be so associated with "white decay" as to account for all of its destructive features. We are all painfully familiar with its whitish appearance, very rapid destruction and sensitive surroundings, and also the difficulty of arresting its progress by filling. Nitric acid is such a powerful solvent that but few substances can stand before it, and both organic and inorganic tooth tissue succumb to its powerful influence. The soluble salts thus formed are promptly removed, exposing fresh surfaces to be displaced in like manner. If it is in concentrated form or considerable quantity, it sweeps with the besom of destruction. If it is more dilute, it more quietly chooses the material for which it has the greatest liking.

Dr. Watt proposed a very plausible theory to account for this dire destruction. Nitric aid can be, and is, formed in the mouth—although some investigators dispute the fact, because they have not found it present in a free state, in which condition it would, as they claim, be general it is destructive, and not confined to the limits of a carious cavity. They lose sight of the fact that it combines, atom by atom, as fast as it is formed (which is its nascent condition) with the elements of tooth-substance.

Nitric acid, as you know, is composed of $N_5 0$, and though these two elements unite in several proportions, this is their favor.te combination—the others readily decomposing and yielding to this.

In the oral cavity, all the conditions necessary in the case are present. All we want is a supply of nitrogen to be oxydized. Oxygen is universal, in the mouth, everywhere. Ammonia, too, is frequently present in the mouth, as can be detected by its odor, and sometimes, too, it is there combined with that most offensive compound, sulphuretted hydrogen. Ammonia is composed of nitrogen and hydrogen, and whenever oxygen is supplied, it is decomposed into water and nitric acid. Wherever there is putrefaction there is ammonia formed, and if to it we can supply oxygen, we have our nitric acid.

Thus we satisfactorily account for the presence of nitric acid, and reasonably explain its "*modus operandi*" in "white decay." It is often, also, administered as a tonic.

Miller disputes the agency of nitric acid in "white decay," because he has found putrifying meats in cavities of decay, and obtained, from tests of the cavities, an alkaline instead of an acid reaction. This is in accordance with our theory. The alkalinity is due to the ammonia in the putrefactive process, which is always in excess—the necessary amount of oxygen being added to a portion of ammonia, an atom of nitric acid is at once formed, and immediately, in its dascent state, combined with the tooth element, leaving no acidiiy to be detected. *Our* nitric is never in excess, and its action is always instantaneous and local.

To lactic and acetic acids is generally assigned the responsibility of destruction in that form of decay known as "chemical abrasion." The animal and earthy portions of the teeth are equally and rapidly acted upom by them, very soluble compounds being formed which are promptly washed away. Hence, the even, smooth, non-colored surface here presented.

These acids are frequently introduced into, and are readily formed within, the mouth, and if concentrated, and undisturbed for a limited time, will produce just what we so often see to our dismay.

It is difficult to explain the location and the peculiar form of this erosion. The labial surfaces and the cutting edges of the incisors are its favorite localities, instead of a general diffusion over the surfaces of all the teeth.

It would seem that the agent must be located, or concentrated, at the exact point of action, and in order to get it in position, I have supposed that it is an acid mucus copiously secreted, and eliminated by the mucous glands of the lip in the one case, and of the tip of the tongue in the other.

Malic, citric, butyric, and other acids, in concentrated form, would effect the teeth by corroding their general surfaces, as in the case of "setting the teeth on edge," and thus predispose them to be acted upon by the mineral acids, favorable located.

Being very soluble acids, they are readily diluted by the saliva, and are not apt, therefore, to act upon definite points. In the case of those sensitive spots about the festoons of the gums, it may be more easily understood that they are the results of acetic acid, formed by fermentation at the point.

Whilst we have treated of these several will-defined types of caries, there are cavities of decay not definitely belonging to any particular one of them, but having some characteristics of the others. So it is not necessary that there should be in each case but a single agent, and it acting continuously. There may be two or more different acids, acting together or in conflict, alternately or intermittingly, producing these blended varieties.

The scope of this paper will not permit an investigation of the process and effects of fermentation, vinous and acetous upon the teeth.—*Southern Dental Journal.*

